# Graded Mobilization With Pacing Technique for Functional Mobility in a Preoperative Marfan Syndrome Case of Aortic Root Dilation: A Case Report

**DOI:** 10.7759/cureus.54591

**Published:** 2024-02-20

**Authors:** Sawari S Bhagwatkar, Vaishnavi Yadav, Prajyot Ankar, Neha Arya

**Affiliations:** 1 Cardiorespiratory Physiotherapy, Ravi Nair Physiotherapy College, Datta Meghe Institute of Higher Education and Research, Wardha, IND

**Keywords:** physical therapy, aortic root dilation, functional mobility, pacing technique, graded mobilization, marfan syndrome

## Abstract

Marfan syndrome (MFS) presents complex cardiovascular manifestations and challenges in management due to its impact on multiple body systems. This case study examines the clinical profile, diagnostic findings, and physiotherapy intervention for a 57-year-old male with MFS who experienced severe aortic and mitral valvular complications. The patient's admission was marked by fatigue, reduced mobility, breathlessness, and a confirmed diagnosis of MFS. Cardiac evaluation revealed severe regurgitation and aortic root dilation. The patient's symptoms were exhaustion, giddiness, dyspnea, and decreased mobility. The objective of this case study was to describe the impact of graded mobilization and pacing techniques in maximizing functional mobility and alleviating symptoms associated with aortic regurgitation and aortic root dilatation through an extensive physiotherapy program. Exercises addressing dyspnea, lung capacity, posture, functional mobility, and fatigue reduction were included in the physiotherapy intervention. The rehabilitation outcome showed a notable shift of score from 3 to 0.5 on the Borg scale of dyspnea, indicating enhanced functional capacity and improved quality of life. Post-rehabilitation, the patient exhibited significant progress in the two-minute walk test. This case highlights the importance of tailored interventions in managing MFS-related cardiac complications.

## Introduction

Marfan syndrome (MFS) is a genetic connective tissue condition that primarily affects the cardiovascular system, skeletal system, ocular system, and skin, among other systems in the body [1]. Because connective tissues play a wide range of roles in the body, people with MFS may be at risk for a number of potentially serious or deadly concurrent medical conditions as a result of the progression of the condition. MFS is caused by an autosomal dominant mutation in the gene encoding the glycoprotein fibrillin-1 (FBN1), which is the major component of microfibrils and has a role in cell anchoring to the extracellular matrix [2]. Pathogenic mutations in FBN1 were discovered as the etiology of MFS in the early 1990s [3-6]. As much as 25 percent of FBN1 pathogenetic variations are de novo, meaning the mutation is novel in the symptomatic patient. Microfibrils play a crucial role in imparting elasticity and strength to connective tissues while also facilitating the release of growth factors essential for tissue expansion and repair throughout the body. In MFS, a genetic mutation leads to reduced production of FBN1, a key component of microfibrils. This scarcity limits the formation of microfibrils, causing significant alterations in the structure of connective tissues and impeding normal tissue growth processes. Essentially, the mutated gene disrupts the intricate balance required for maintaining the integrity and functionality of connective tissues, contributing to the diverse manifestations observed in MFS [7]. Aortic root aneurysm, acute aortic dissection, disproportionate long bone overgrowth, and ectopia lentis (the displacement or malposition of the crystalline lens of the eye) are the most common multifaceted signs of MFS. It is a highly penetrating syndrome with significant intrafamilial and interfamilial heterogeneity. MFS impacts around one in every 5000 persons and is equally distributed among males and females, as well as religions and ethnicities [8].

MFS is characterized by its most prominent physical features, which encompass an elongated physique characterized by a slender build with extended fingers, arms, and legs. As it is said that MFS primarily affects the cardiovascular system, one of the primary clinical presentations of MFS centers around aortic complications [9]. In the majority of MFS patients, the thoracic aortic ailment commences as an asymptomatic dilation of the aortic root, gradually expanding over time to form an aneurysm - a structural weakening of the arterial wall resulting in a conspicuous bulging or distension. While medications can decelerate the rate of this enlargement, they are unable to forestall its progression entirely [9]. The aortic aneurysm, as it continues to expand, becomes increasingly unstable and may eventually precipitate an acute ascending aortic dissection, commonly referred to as type A dissection according to the Stanford classification. This condition represents a life-threatening complication of MFS and can substantially diminish lifespan [9]. A fresh pleural effusion or pericardial effusion noticed on radiographs might additionally indicate the presence of aortic dissection [10]. The dilatation of the aortic root, the aorta segment nearest to the heart, is the most common aortic event linked with MFS.

At the start of disease progression, aortic root dilatation is usually symmetrical and localized to the aortic root. Aortic root dilatation is diagnostic, but the typical aortic root diameter must be corrected using nomograms based on age, gender, height, and weight [11]. Aortic root dilation results in aortic regurgitation characterized by a central jet, which occurs due to the dilation of the aortic annulus and the degeneration of the aortic valve with a myxomatous nature [12]. The volume of blood expelled with each left ventricle (LV) contraction increases as the regurgitant volume increases. To sustain cardiac output, the end-diastolic LV volume expands, and the left atrium expands, reflecting the increased end-diastolic LV pressure and left atrial pressure. Due to adaptive rises in ventricular contraction, the LV ejection fraction does not drop. Chronic volume overload can cause myocardial injury and the failure of compensatory mechanisms, causing cardiac output to fall. Myocardial injury induces global hypokinesis by affecting the whole LV [13]. Patients with aortic root dilation and aortic regurgitation often undergo surgical management for aortic valve repair or aortic valve replacement. Shortness of breath leads to reduced functional capacity; hence, it is suggested to avoid heavy physical activities. Due to shortness of breath, the patient did suffer from fatigue, restricted mobility, and fear of mobility. Pacing enables people to control how well they use their energy resources and how exhaustion affects their exercise performance [14]. Graded mobilization reduces the chance of injury and overexertion by being gradual. It offers patients a safer way to improve mobility without pushing them above their existing limits by gradually increasing the intensity and complexity of activities.

## Case presentation

A 57-year-old male presenting with Marfanoid features sought medical attention at the hospital with complaints of reduced mobility, breathlessness, fatigue, and giddiness persisting for the past three months. His breathlessness had a gradual onset and was assessed using the Borg scale of dyspnea, with a score of 3. The patient disclosed a history of similar symptoms four years prior, which had been relieved through medication at that time. During the current hospitalization, diagnostic tests were conducted, including a 2D echocardiogram and electrocardiography (ECG). The patient's clinical presentation aligns with the criteria established by the Ghent criteria, confirming the diagnosis of MFS. For the above complaints, the patient was referred to a cardiorespiratory physiotherapy program for further management.

Clinical findings

**Figure 1:**
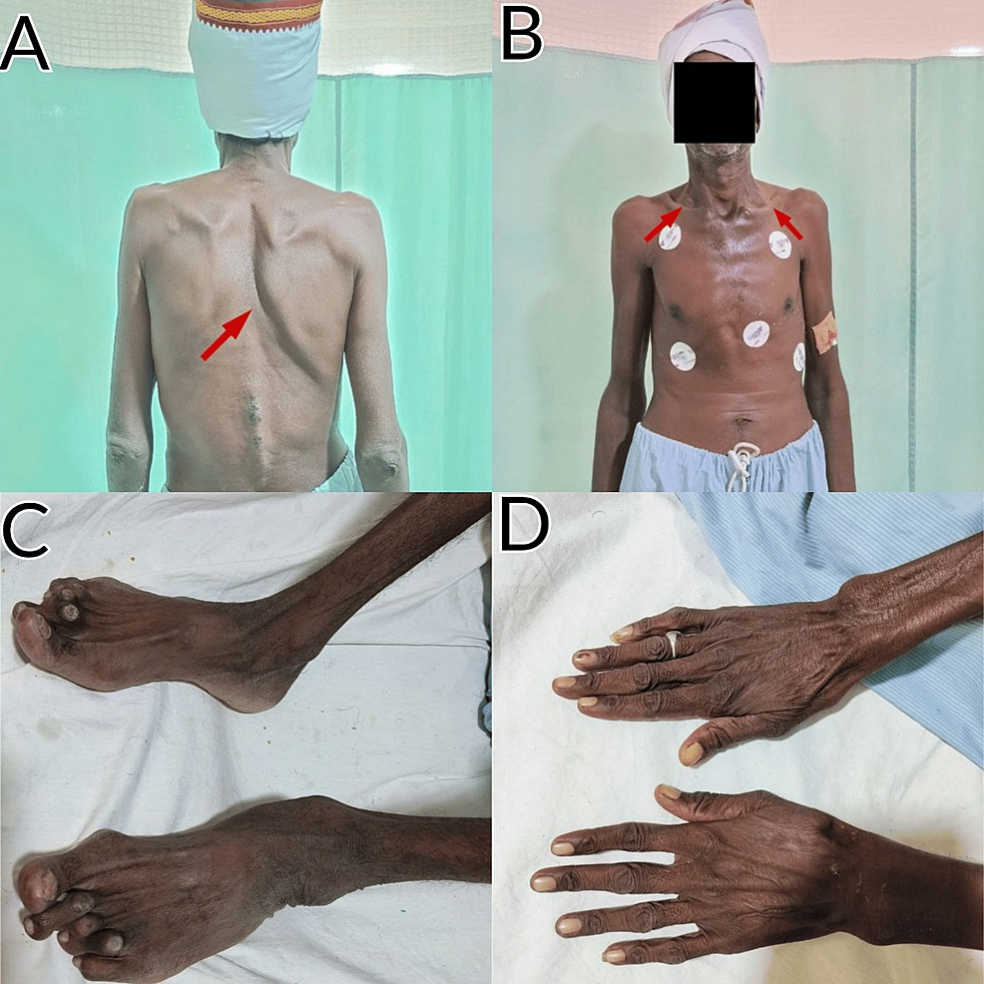
Physical features observed in the patient (A) Scoliosis towards the left, upward and outward rotation of the scapula (B) Supraclavicular hollowness bilaterally and pectus carinatum (C) Calluses on toes (D) Elongated hands with long and thin fingers

The patient's oral consent was taken before the examination, and the patient was conscious and well aware of person, place, and time. Upon observation, the patient exhibited a hammer-toe appearance, elongated feet, and lower limbs. Calluses were also noted. There was scoliosis towards the left, extended hands with long and slender fingers, and muscle wasting in the right lower limb. In the chest, bilateral supraclavicular hollowness and pectus carinatum were noted. Additionally, the patient displayed the use of accessory muscles and difficulty in breathing. On examination, the Borg scale of dyspnea indicated breathlessness with a score of 3. Figure 1 depicts different physical features observed in the patient.

Clinical diagnosis

**Figure 2:**
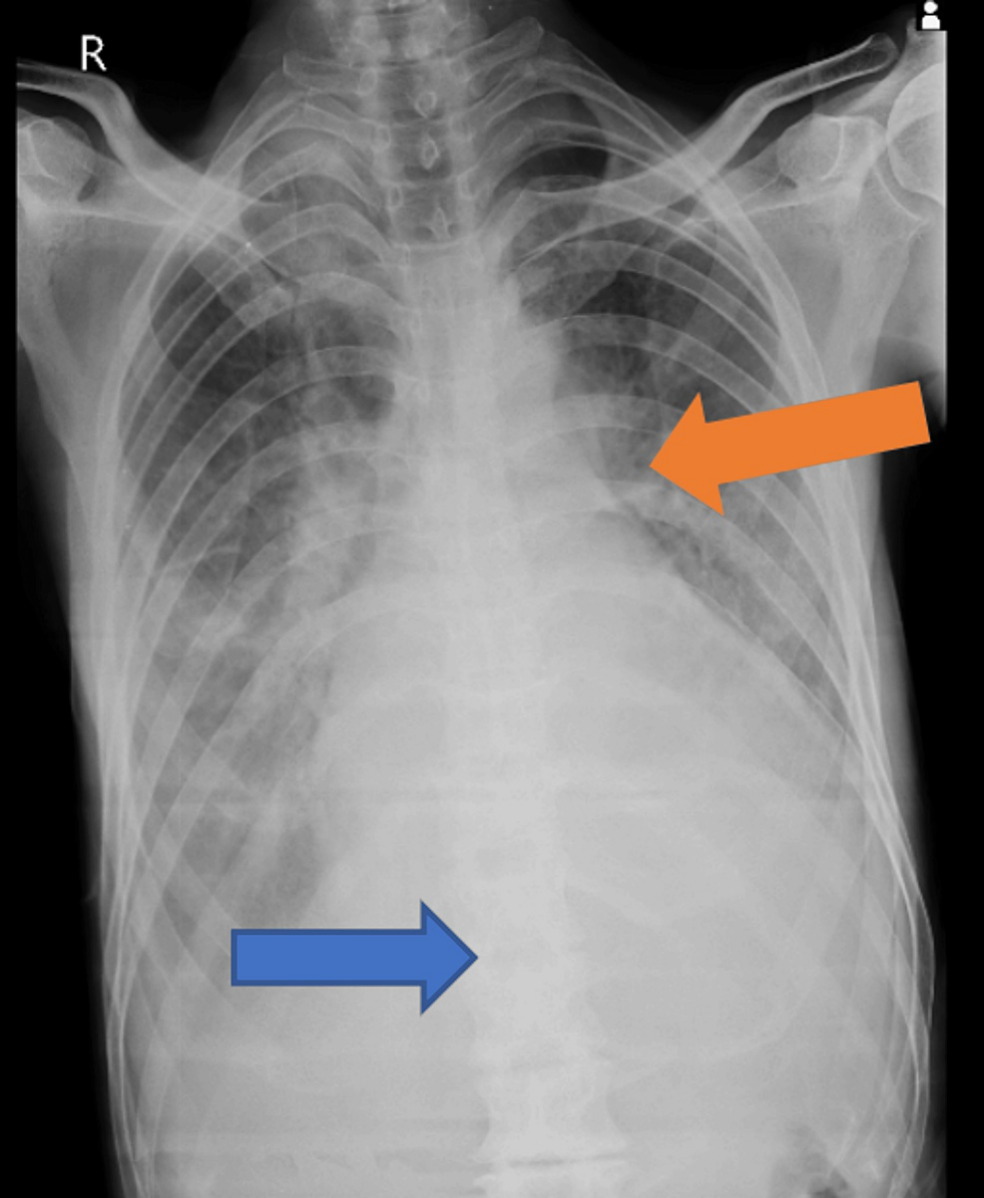
Chest X-ray of the patient Homogenous opacity on left lower zone (blue arrow) and left ventricular hypertrophy (orange arrow)

Diagnostic procedures, such as an ECG, were done, which showed a large S wave indicating valvular heart disease. A 2D echocardiogram was also performed, which revealed that the patient had severe aortic regurgitation involving a trileaflet valve, anterior mitral leaflet (AML) prolapse accompanied by severe mitral regurgitation, mild pericardial effusion, and a dilated left atrium and ventricle. A chest X-ray demonstrated homogenous opacity on the left lower zone and left ventricular hypertrophy. Figure 2 depicts the chest X-ray of the patient.

Physiotherapy intervention

The patient was provided with preoperative physiotherapy intervention for two weeks. Physiotherapy intervention was given for proper and early recovery. The physiotherapy intervention is described in Table 1.

**Table 1:** Physiotherapy intervention

Goals	Physiotherapy intervention	Sets
To reduce shortness of breath at rest	Medical management: pursed lip breathing	10 repetition × 1 set
To improve lung volume and capacity	Incentive spirometry, segmental breathing exercises, and thoracic expansion exercises	10 repetition × 1 set
To improve posture	Patient education on posture, chest mobilization, and side stretching from the concave side	10 repetition × 1 set
To enhance functional mobility	Mobilization: active range of motion exercises for bilateral upper and lower limbs. In-bed exercises: ankle pump, heel slides, bed-side sitting, and ambulation	10 repetition × 1 set
To reduce fatigue	Pacing techniques: pacing involves cutting up activity into manageable chunks to reduce exertion	10 repetition × 1 set

**Figure 3:**
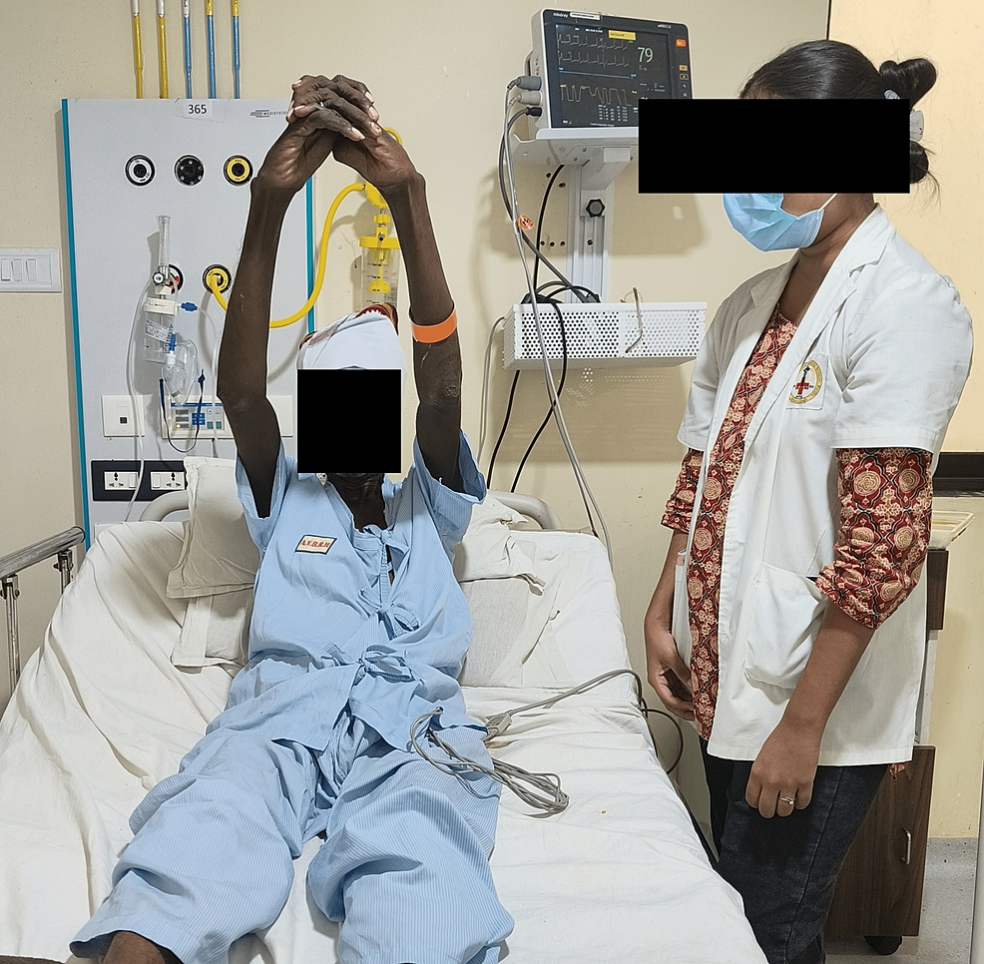
Thoracic expansion exercises being performed by the patient

Figure 3 shows the patient performing thoracic expansion exercises.

Outcome measures

The Borg scale of dyspnea (validity: 0.84, reliability: 0.85) was used to assess breathlessness, the quality of life scale (validity: 0.79, reliability: 0.89) to assess the impaired activities of individuals' day-to-day life, the two-minute walk test (validity: 0.88, reliability: 0.95) to check endurance, chest expansion to measure expansion capacity of the lung, and the fatigue severity score (validity: 0.69, reliability: 0.89) to assess fatigue. The Borg scale of dyspnea showed a significant improvement from a pre-rehabilitation score of 3 to a post-rehabilitation score of 0.5, while the quality of life increased from 60 to 85. The fatigue severity score also showed improvement, decreasing from 56 to 32. The two-minute walk test, a reliable measure of functional exercise capacity, indicated a substantial improvement with an increase from 80 m pre-rehabilitation to 120 m post-rehabilitation. The outcome measures for pre-rehabilitation (on day 1) and post-rehabilitation (after two weeks) are given in Table 2.

**Table 2:** Outcome measures for pre-rehabilitation and post-rehabilitation [15,16]

Outcome measure	Pre-rehabilitation	Post-rehabilitation
Borg scale of dyspnea	3	0.5
Quality of life	60	85
Two-minute walk test	80 m	120 m
Chest expansion	Axillary level: 1 cm; Xiphisternum level: 2 cm	Axillary level: 2 cm; Xiphisternum level: 3 cm
Fatigue severity score	56	32

## Discussion

In this case report, graded mobilization and pacing technique was used as a physiotherapy treatment for a 57-year-old male with MFS, along with aortic root dilatation preoperatively, which enhanced functional mobility and quality of life of the individual. The accomplishment of goals was positively impacted by energy conservation through interval exercise training and pacing. Aortic root dilatation presents special problems that call for a sophisticated approach that takes into account not just cardiovascular factors but also the significance of graded progression and pacing in rehabilitation. Gradual mobilization guarantees a methodical and customized progression of exercises, considering the unique requirements and constraints of each patient. The physiotherapy intervention for the patient with aortic regurgitation and aortic root dilatation was designed to optimize respiratory function and cardiovascular health. Thus, a key component of the all-encompassing care of MFS patients with aortic root dilatation is the combination of graded mobilization with pacing technology, which provides a route to better preoperative functional results. The case study presents a physiotherapy intervention program designed to address various aspects of the patient's respiratory condition. The program focuses on shortness of breath, reduced lung volume, compromised posture, limited functional mobility, and persistent fatigue [17].

Thoracic extension and rotation are expected to be improved by chest wall mobilization during rehabilitation, leading to a decrease in the lower thoracic excursion. Because of this notable increase in chest expansion capacity, respiratory muscles can contract at their ideal length, increasing their strength. Pursed lip breathing techniques were skillfully employed to alleviate breathlessness, while thoracic expansion exercises were introduced to optimize air entry into all segments of the lungs, which is crucial for effective respiration [18]. An uncommon set of clinical findings, including severe aortic regurgitation, aortic root dilatation, exhaustion, decreased movement, and dyspnea is described in this case report. It is remarkable that a single patient may have such a constellation of symptoms, as it gives professionals insight into potential difficulties with diagnosis and treatment and how effectively it can be managed through physiotherapy. One distinctive feature of this case is the physiotherapy intervention's incorporation of graded mobilization and pacing strategies [19].

The intervention's effectiveness is measured through a comprehensive set of outcome measures. The structured rehabilitation program proved instrumental in yielding these remarkable outcomes, effectively blending tailored physiotherapy interventions, comprehensive patient education, and a gradual but progressive increase in physical activity. The strategic emphasis on personalized education regarding coronary artery disease (CAD) risk factors, pacing activities, and the use of appropriate exertion scales notably contributed to the holistic success of the intervention. In essence, the intervention's structured approach, encompassing preoperative physiotherapy, underscores the pivotal role of physiotherapy in optimizing functional capacity, symptom management, and enhancing the overall quality of life for patients with aortic regurgitation and aortic root dilatation [20].

## Conclusions

This intervention is a structured and graduated approach that underscores the pivotal contribution of physiotherapy in enhancing the well-being, functional capacity, and symptom management of individuals grappling with aortic regurgitation and aortic root dilatation along with MFS. This conclusion encapsulates the positive outcomes and emphasizes the significant role of physiotherapy in improving the functional capacity and overall well-being of individuals with MFS associated with aortic regurgitation. The patient's symptoms were effectively managed, and their functional mobility was maximized with the use of a thorough physiotherapy strategy that combined pacing techniques with graded mobilization. The research highlights the significance of customized therapies in the management of cardiac problems associated with MFS. Improvements in quality of life, decreased severity of fatigue, increased functional exercise capacity, and a discernible shift in the Borg dyspnea scale were among the major outcome measures that showed improvements as a result of the organized rehabilitation program. Because of the peculiar combination of symptoms, the comprehensive physiotherapy intervention that focuses on patient-centered care, and the thorough description of clinical and genetic factors, this case report stands out as a significant contribution to the medical literature. It not only improves our knowledge of cardiac issues associated with MFS but also offers practical advice to medical professionals managing instances that are comparable to it.
